# Supplementary material for: Comparison of Antibiotic Resistance Mechanisms in Antibiotic-Producing and Pathogenic Bacteria
Source: Molecules. 2019 Sep 21;24(19):3430. doi: 10.3390/molecules24193430 (PMC6804068; doi:10.3390/molecules24193430)
Supplement: Supplementary file 1 [file molecules-24-03430-s001.zip › Figure S1.docx]

1000

901

1000

958

1000

990

947

832

1000

1000

976

583

1000

1000

703

1000

1000

804

1000

1000

795

997

974

679

1000

1000

995

874

1000

1000

1000

A

B

C

D

E

Figure S1. Phylogenetic tree of aminoglycoside acetyltransferases on the basis of amino acid sequences of those from antibiotic producers and pathogens. The tree was constructed by using ClustalX2 as described previously [5]. GenBank accession numbers and derived bacterial species are shown in the figure. A, B, C, D and E indicate cluster numbers. The bootstrap probabilities are shown at branching nodes. The antibiotic producers are marked with red square.
