# Supplementary material for: Comparison of Antibiotic Resistance Mechanisms in Antibiotic-Producing and Pathogenic Bacteria
Source: Molecules. 2019 Sep 21;24(19):3430. doi: 10.3390/molecules24193430 (PMC6804068; doi:10.3390/molecules24193430)
Supplement: Supplementary file 1 [file molecules-24-03430-s001.zip › Figure S2.docx]

A


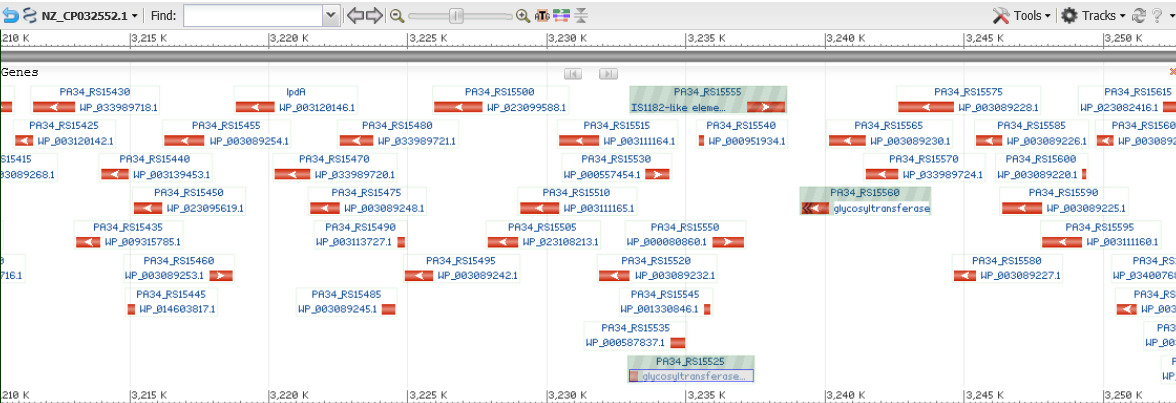


| PA34_RS15475: 2-oxoisovalerate dehydrogenase subunit beta, 350aa | PA34_RS15535: hypothetical protein, 180aa |
| --- | --- |
| PA34_RS15480: 3-methyl-2-oxobutanoate dehydrogenase (2-methylpropanoyl-transferring) subunit alpha, 410aa | PA34_RS15540: hypothetical protein, 63aa |
| PA34_RS15485: Bkd operon transcriptional regulator, 153aa | PA34_RS15545: hypothetical protein, 81aa |
| PA34_RS15490: inner centromere protein, 97aa | PA34_RS15550: DUF3883 domain-containing protein, 378aa |
| PA34_RS15495: DNA topoisomerase IB, 333aa | PA34_RS15555: Pseudo, IS1182-like element ISCfr1 family transposase, 1371nt |
| PA34_RS15500: FAD-dependent oxidoreductase, 577aa | PA34_RS15560: Pseudo, Partial stop, glycosyltransferase, 960nt |
| PA34_RS15505: acyltransferase family protein, 355aa | PA34_RS15565: biofilm formation protein PslG, 442aa |
| PA34_RS15510: membrane protein, 469aa | PA34_RS15570: glycosyltransferase, 395aa |
| PA34_RS15515: biofilm formation protein PslJ, 478aa | PA34_RS15575: biofilm formation protein PslE, 662aa |
| PA34_RS15520: glycosyltransferase family 1 protein, 367aa | PA34_RS15580: polysaccharide export protein, 256aa |
| PA34_RS15525: Pseudo, Partial start, glycosyltransferase family 1 protein, 279nt | PA34_RS15585: glycosyltransferase family 2 protein, 303aa |
| PA34_RS15530: aminoglycoside N-acetyltransferase AAC(3)-IId, 286aa |  |

B


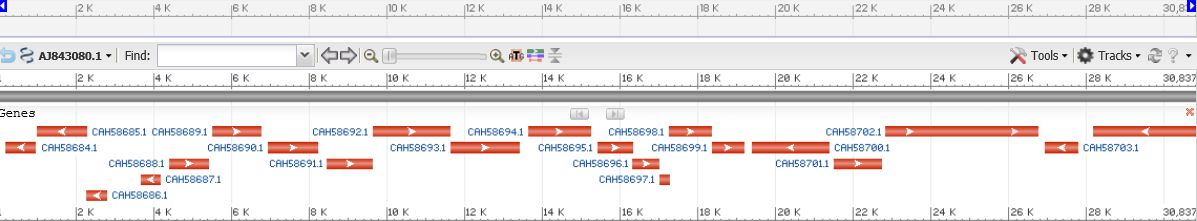


| CAH58704.1: putative LuxR regulator, 886aa |
| --- |
| CAH58703.1: putative aminoglycoside N-acetyltransferase, 287aa |
| CAH58702.1: hypothetical protein, 1313aa |
| CAH58701.1: putative aminomutase, 416aa |
| CAH58700.1: putative ribosyltransferase, 660aa |
| CAH58699.1: hypothetical protein, 279aa |
| CAH58698.1: hypothetical protein, 366aa |
| CAH58697.1: hypothetical protein, 83aa |
| CAH58696.1: putative phosphomutase, 233aa |
| CAH58695.1: putative Fe-S oxidoreductase, 299aa |
| CAH58694.1: putative sugar dehydrogenase, 541aa |
| CAH58693.1: putative exporter, 594aa |
| CAH58692.1: putative exporter, 663aa |
| CAH58691.1: putative glycosyltransferase, 394aa |
| CAH58690.1: putative 2-deoxy-scyllo-inosose synthase, 430aa |

Figure S2. The gene arrangements around the aminoglycoside N-acetyltransferases in *Pseudomonas aeruginosa* PA34 (A) and *Streptomyces fradiae* (B). The figures are redrawn from the NCBI database. The GenBank accession numbers are NZ_CP032552.1 and AJ843080.1, respectively.
