# Supplementary material for: Comparison of Antibiotic Resistance Mechanisms in Antibiotic-Producing and Pathogenic Bacteria
Source: Molecules. 2019 Sep 21;24(19):3430. doi: 10.3390/molecules24193430 (PMC6804068; doi:10.3390/molecules24193430)
Supplement: Supplementary file 1 [file molecules-24-03430-s001.zip › Figure S3.docx]

1000

850

1000

666

1000

616

839

996

776

1000

988

1000

709

1000

1000

995

804

1000

876

A

B

C

Figure S3. Phylogenetic tree of aminoglycoside phosphotransferases on the basis of amino acid sequences of those from antibiotic producers and pathogens. The tree was constructed by using ClustalX2 as described previously [5]. GenBank accession numbers and derived bacterial species are shown in the figure. A, B and C indicate cluster numbers. The bootstrap probabilities are shown at branching nodes.　The antibiotic producers are marked with red square.
