# Supplementary material for: Comparison of Antibiotic Resistance Mechanisms in Antibiotic-Producing and Pathogenic Bacteria
Source: Molecules. 2019 Sep 21;24(19):3430. doi: 10.3390/molecules24193430 (PMC6804068; doi:10.3390/molecules24193430)
Supplement: Supplementary file 1 [file molecules-24-03430-s001.zip › Figure S4.docx]

1000

1000

815

554

1000

1000

677

997

768

1000

1000

998

858

719

871

1000

1000

1000

A

1000

B

C

Figure S4. Phylogenetic tree of aminoglycoside rRNA methyltransferases on the basis of amino acid sequences of those from antibiotic producers and pathogens. The tree was constructed by using ClustalX2 as described previously [5]. GenBank accession numbers and derived bacterial species are shown in the figure. A, B and C indicate cluster numbers. The bootstrap probabilities are shown at branching nodes.
