# Supplementary material for: Comparison of Antibiotic Resistance Mechanisms in Antibiotic-Producing and Pathogenic Bacteria
Source: Molecules. 2019 Sep 21;24(19):3430. doi: 10.3390/molecules24193430 (PMC6804068; doi:10.3390/molecules24193430)
Supplement: Supplementary file 1 [file molecules-24-03430-s001.zip › Figure S5.docx]

1000

1000

1000

526

1000

743

422

981

1000

1000

1000

988

864

1000

938

743

1000

359

1000

527

1000

999

A

1000

B

C-1

C-2

C-3

C-4

D

Figure S5. Phylogenetic tree of aminoglycoside transporters on the basis of amino acid sequences of those from antibiotic producers and pathogens. The tree was constructed by using ClustalX2 as described previously [5]. GenBank accession numbers and derived bacterial species are shown in the figure. A, B, C and D indicate cluster numbers. The bootstrap probabilities are shown at branching nodes.　The pathogenic bacteria are marked with blue square.
