# Supplementary material for: Comparison of Antibiotic Resistance Mechanisms in Antibiotic-Producing and Pathogenic Bacteria
Source: Molecules. 2019 Sep 21;24(19):3430. doi: 10.3390/molecules24193430 (PMC6804068; doi:10.3390/molecules24193430)
Supplement: Supplementary file 1 [file molecules-24-03430-s001.zip › Figure S6.docx]

A

958

1000

1000

884

979

370

525

1000

1000

1000

B

Figure S6. Phylogenetic tree of aminoglycoside transporters on the basis of amino acid sequences of those from antibiotic producers and pathogens. The tree was constructed by using ClustalX2 as described previously [5]. GenBank accession numbers and derived bacterial species are shown in the figure. A and B indicate cluster numbers. The bootstrap probabilities are shown at branching nodes. The antibiotic producers are marked with red square.
