# Supplementary material for: Comparison of Antibiotic Resistance Mechanisms in Antibiotic-Producing and Pathogenic Bacteria
Source: Molecules. 2019 Sep 21;24(19):3430. doi: 10.3390/molecules24193430 (PMC6804068; doi:10.3390/molecules24193430)
Supplement: Supplementary file 1 [file molecules-24-03430-s001.zip › Figure S7.docx]

1000

628

1000

793

725

1000

988

999

1000

801

1000

1000

Figure S7. Phylogenetic tree of carbapenemases from pathogens and some β-lactamases from *Streptomyces* species on the basis of their amino acid sequences. The tree was constructed by using ClustalX2 as described previously [5]. GenBank accession numbers and derived bacterial species are shown in the figure. The bootstrap probabilities are shown at branching nodes. Class C β-lactamase from *Actinosynnema mirum* (Amir_3080) was used as outgroup.　The antibiotic producers are marked with red square.
